# Health priority-setting for official development assistance in low-income and middle-income countries: a Best Fit Framework Synthesis study with primary data from Ethiopia, Nigeria and Tanzania

**DOI:** 10.1186/s12889-021-12205-6

**Published:** 2021-11-21

**Authors:** Xiaoxiao Jiang Kwete, Yemane Berhane, Mary Mwanyika-Sando, Ayo Oduola, Yuning Liu, Firehiwot Workneh, Smret Hagos, Japhet Killewo, Dominic Mosha, Angela Chukwu, Kabiru Salami, Bidemi Yusuf, Kun Tang, Zhi-Jie Zheng, Rifat Atun, Wafaie Fawzi

**Affiliations:** 1 Harvard TH Chan School of Public Health, 677 Huntington Avenue, Boston, MA 02150 USA; 2 Addis Continental Institute of Public Health, Addis Ababa, Ethiopia; 3 Africa Academy for Public Health, Dar es Salaam, Tanzania; 4 University of Ibadan Research Foundation, Ibadan, Nigeria; 5 JPMorgan Chase Institute, Washington, DC, USA; 6 Muhimbili University of Health and Allied Sciences, Dar es Salaam, Tanzania; 7 Tsinghua University Vanke School of Public Health, Beijing, China; 8 Peking University School of Public Health, Beijing, China

**Keywords:** Priority setting, Official development assistance, Qualitative case study, Health systems

## Abstract

**Background:**

Decision making process for Official Development Assistance (ODA) for healthcare sector in low-income and middle-income countries involves multiple agencies, each with their unique power, priorities and funding mechanisms. This process at country level has not been well studied.

**Methods:**

This paper developed and applied a new framework to analyze decision-making process for priority setting in Ethiopia, Nigeria, and Tanzania, and collected primary data to validate and refine the model. The framework was developed following a scoping review of published literature. Interviews were then conducted using a pre-determined interview guide developed by the research team. Transcripts were reviewed and coded based on the framework to identify what principles, players, processes, and products were considered during priority setting. Those elements were further used to identify where the potential capacity of local decision-makers could be harnessed.

**Results:**

A framework was developed based on 40 articles selected from 6860 distinct search records. Twenty-one interviews were conducted in three case countries from 12 institutions. Transcripts or meeting notes were analyzed to identify common practices and specific challenges faced by each country. We found that multiple stakeholders working around one national plan was the preferred approach used for priority setting in the countries studied.

**Conclusions:**

Priority setting process can be further strengthened through better use of analytical tools, such as the one described in our study, to enhance local ownership of priority setting for ODA and improve aid effectiveness.

## Background

Official Development Assistance (ODA) is an important source of health financing in many low-income and middle-income countries (LMICs) [1–3]. Although most LMICs have been able to achieve the Millennium Development Goals (MDGs) with the support of the global community, studies examining the association between improvements in MDG indicators and ODA have yielded mixed results [4–7].

Given the large amount of ODA funding and its uncertain effects on development, it is important to understand how ODA is applied, to ensure that scarce resources are used efficiently in addressing the most pressing health needs in LMICs. This process is referred to as priority setting throughout this study. Since the Paris Declaration [8], Accra Agenda for Action [8], and subsequent international agreements relating to ODA, the global development community has reached a consensus that local ownership and ODA alignment to national strategies and plans are key to sustainable development. Nonetheless, there is a persistent tension between donor countries providing ODA and governments of recipient countries on how ODA should be allocated. Priority setting for health in recipient countries is still heavily influenced by donor interests and, in some cases, donor-driven [9–13]. The power asymmetry between the donors and recipient countries can explicitly or implicitly impact the behavior of institutions receiving ODA [14, 15].

The Paris Declaration has prompted the development of different standards and initiatives to change donor behavior to respect local ownership and better align ODA to local priorities - including Sector Wide Approaches (SWAPs) and the International Health Partnership Plus (IHP+). However, studies examining changes in donor behavior have revealed mixed outcomes on achieving the goals set in the Paris Declaration [16–19].

Earlier studies [20–24] have examined the systematic approaches used by donor countries to set their priorities and influence the agenda of recipient governments, and different frameworks have been proposed by academic scholars to study these approaches [25]. However, few studies have examined priority setting processes in recipient countries from the perspective of local governments to ascertain how they align or differ from the priorities identified by bilateral and multilateral agencies, as well as scholars.

Policy formulation involves intricate processes that results-focused and purely quantitative evaluation studies fail to account for or capture [26–29]. We view this process to be more aligned with that of a complex open system, and thus can be better explained by the use of system theory. Although system theory did not originate from health system analysis [30, 31], many scholars have attempted to apply it to this area [32]. This study was designed with the same line of thought that decision making for priority setting in the health sector has the same characteristic as a complex system, that it is comprised of a set of interconnected elements, it is more than a simple additive of functions of the components, the process is non-linear and the results are often unpredictable and can be counterintuitive [33–35].

This study aimed to answer the question of what mechanisms, tools and processes are used by recipient countries in determining resource allocation for ODA for health, from the perspective of local stakeholders. Based on a scoping review undertaken as part of this study, the authors developed and tested an analytical framework using field studies in Ethiopia, Nigeria and Tanzania for examination and comparative assessment of priority setting process in relation to ODA to ascertain donor and local influences. In this paper ODA for health is used interchangeably as “external funding for health”, and organizations issuing them are referred to as “donors”.

## Methods

### Study design

A Best Fit Framework Synthesis was performed [36], by first building a conceptual framework – the a priori framework - from a scoping review using the Arksey and O’Mally method [37] and then testing it against primary data collected from three case countries.

For the scoping review and framework development, relevant published articles were identified from PubMed, Embase and Web of Science (Thomson) using the following search terms: 1) low- and middle-income countries, 2) external funding and aid, 3) health, and 4) tools and processes. General terms such as “tools” and “processes” were used, instead of “priority setting” or “agenda setting”, to include articles which described the process of using external funding for health but didn’t specifically use the term “priority setting”. Two researchers (XK and YL) independently reviewed the abstracts of the articles retrieved by the search and selected those which were relevant to the research topic and extracted keywords. Differences full-text selection and data extraction were resolved with internal discussions.

Conventional qualitative content analysis and grounded theory method [38–40] were used to analyze the data extracted from the final selection of articles. Keywords emerged from those articles were compared and synthesized into major thematic categories to develop the final analytical framework.

In parallel to the framework development, primary data were collected from Ethiopia, Nigeria, and Tanzania on decision making in external funding for health to be tested against the a priori framework. The three countries were selected from the seven African country members of the China Harvard Africa Network, namely, Botswana, Ethiopia, Ghana, Nigeria, South Africa, Tanzania, and Uganda, based on existing research collaborations and the willingness of the countries to participate in the study.

The interview guide was developed and validified with each country team, and it included general open questions such as “How is your organization involved in setting agenda and priorities for external funding for health?” and “What are the tools/methods/mechanisms used in the process?”. All interviewers were encouraged to use prompt to probe further for detailed description of these tools, methods, and mechanisms relating to decision making for priority setting and to ascertain the origins of the tools, methods, and mechanisms used.

The interviews were initially conducted with key informants who worked in the main department(s) or organization(s) that managed and oversaw external funds, as decided by the research team in each case country. Snowballing methodology was used to identify and interview additional key informants in all departments and organizations involved in priority setting process. These additional key informants were identified by interviewees of the first round of interviews.

All interviews were conducted in person from February 2018 to April 2019 across the three countries. The interviews in Ethiopia were conducted in Amharic by public health researchers fluent in speaking, reading, and writing in Amharic. The interviews in Nigeria were conducted in English and in Tanzania, Swahili.

For those who consented to audio recording, the recording was transcribed in full text. For those who did not give consent to audio recording, meeting notes were taken contemporaneously by the research team undertaking interviews in the respective country. The research teams in Ethiopia and Tanzania are comprised of members fluent in Amharic and Swahili respectively, and they all participated in the coding and interpretation of the data.

In Nigeria, a state was selected for interviews at sub-national government departments, as the decision-making process in Nigeria for prioritizing ODA was de-centralized to state level, as assessed by the local research team.

### Data analysis and synthesis

A framework was developed by the authors using the results of the scoping literature review. Qualitative data from interviews was coded using the layers and themes presented in the framework following conventional qualitative content analysis [38] method: frequencies of keywords that correspond to each of the layers of themes in the framework, in the format of verbatim quotations from study participants or notes from the interviewers, were documented and the context they appeared in were analyzed and compared across themes, layers and countries to identify strength or weakness areas.

## Results

### Scoping review and framework development

**Fig. 1:**
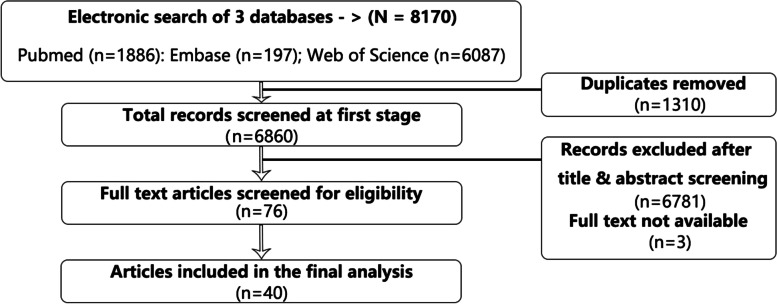
PRISMA chart of the scoping review

The systematic scoping review yielded 8170 results across three databases (1886 from Pubmed, 197 from Embase, and 6087 from Web of Sciences). The search was completed on September 10th, 2018. After removal of 1310 duplicates, 6860 distinct records were retrieved for review following the criteria specified above and generated 79 articles for full-text review. Data extraction and narrative synthesis was conducted on a final list of 40 articles (Fig. 1).

All 40 articles described the decision making process of an LMIC government agency on the use of external funds and 15 of them also described how they resolved disagreements with ODA donors. Thirty-five (35) of them covered a single LMIC and 5 covered multiple countries. Of the 35 articles that covered single country cases, 25 (71%) of the studies were in Africa, 8 (23%) in Asia and 2(6%) in Latin America.

**Fig. 2:**
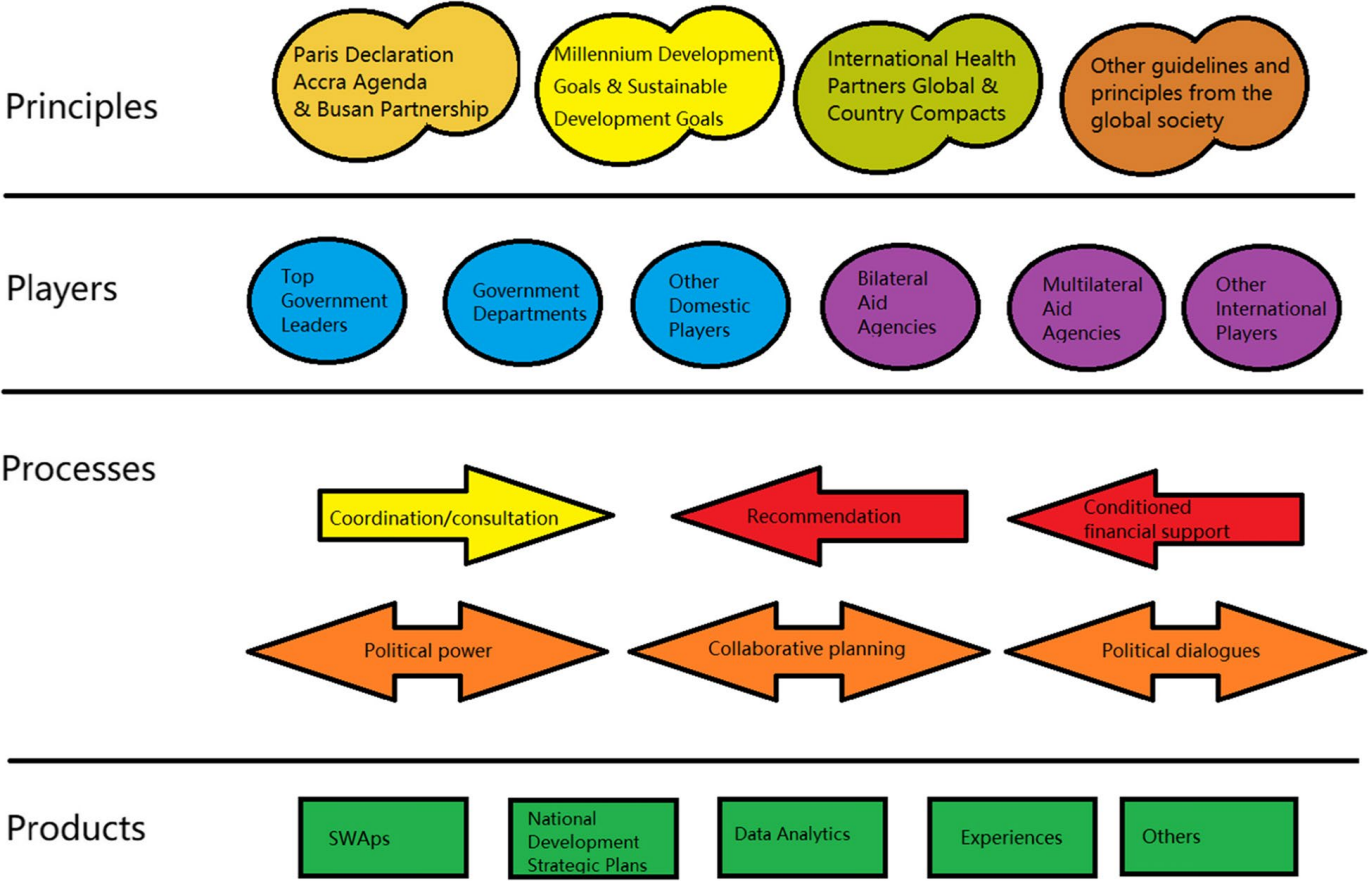
Framework for priority setting of Official Development Assistance at country level. Note: The arrows in Processes suggests the direction of influence. Coordination/consultation is used by the local government to solicit opinions, while recommendation and conditioned financial support are influences from outside of the government. Political power, collaborative planning and political dialogues can work in both ways

Close to 20 sub-themes were identified from the analysis of data emerging from the review relating to elements and actions that influenced decision making for priority setting for ODA. These sub-themes included “data”, “evidence”, “successes from pilot projects”, “consultation of external experts”, “the top leader’s will” and etc. Following the review of the emerging sub-themes, the team categorized them further into four higher themes, namely, Principles, Players, Processes, and Products, each relating to the process of priority setting. Based on the layers of themes identified, we developed a framework for analyzing the priority setting process in LMICs. The framework consists of four inter-related elements: 1) Principles, which shape the global, regional and local contexts (such as Paris Principles, Accra Agenda for Action, and IHP+ which has informed the development of country compacts), 2) Players, which refer to all the entities and individuals who contribute to the decisions made, 3) Processes, which describe the different channels and approaches those players take in exerting their decision making or advisory power, and 4) Products, which represent a variety of tools and instruments developed over the past years that are used by players to shape priority setting process and channeling of funds, such as SWAPs, Sector Investment Plans (SIPs), Budget Support, National Health Plans, National Strategies, disease specific plans, international evidence, and among others, analytic tools, for example One Health Tool (see Fig. 2).

### Interview data

A total of 21 in-depth interviews were conducted (6 in Ethiopia, 10 in Nigeria and 5 in Tanzania), with participants who were holding key positions in different institutions involved in the decision-making process at the time of the interview.

The three countries varied substantially in their economic and demographic status, as well as their levels of reliance on external funding for health. Tanzania has the highest percentage of current health expenditure financed from external sources (32%), followed by Ethiopia (22%) and then Nigeria (8%), see Table 1.

**Table 1:** Key socio-economic, demographic and institutional information about Ethiopia, Nigeria, and Tanzania

	Ethiopia	Nigeria	Tanzania
Income class	Low Income	Lower-Middle Income	Low Income
GDP per capita (current $, 2018)	772.3	2028.20	1061.00
Population size (2018)	109,224,560	195,874,740	56,318,350
External funding for health as a % of current health expenditure (2017)	22.11	7.91	31.82
Number of interviews conducted	7	10	5
Name of institutions interviewed	Federal Ministry of Health	Ministry of Women Affairs and Social Development	Ministry of Health
	Ministry of Finance and Economic Development	Oyo State Ministry of Health	Ministry of Finance and Planning
		Oyo State Primary Health Care Board	President Office-Regional and Local Government Authority (PORALG)
		Oyo State Health Insurance Agency	National AIDS Control Program
		Tuberculosis and Leprosy Control Programme for Oyo State	National TP & Leprosy Program

Source of Data: The World Bank Database, https://data.worldbank.org/, the most recent year was applied

### Contextual features in Ethiopia, Nigeria and Tanzania

Each of the 4 layers of framework - principles, players, processes, and products – informs the analysis of the priority setting process from a different angle. Keywords deemed relevant to each theme were used to search through the transcripts or interview notes. The context, where the keywords were mentioned was analyzed to identify how and where in the decision-making process a particular theme played a role.

Table 2 illustrates how each layer of the framework played a role in the priority setting process. Factors with major influence were marked in green, while factors with minor or no influence were marked in red.

**Table 2:**
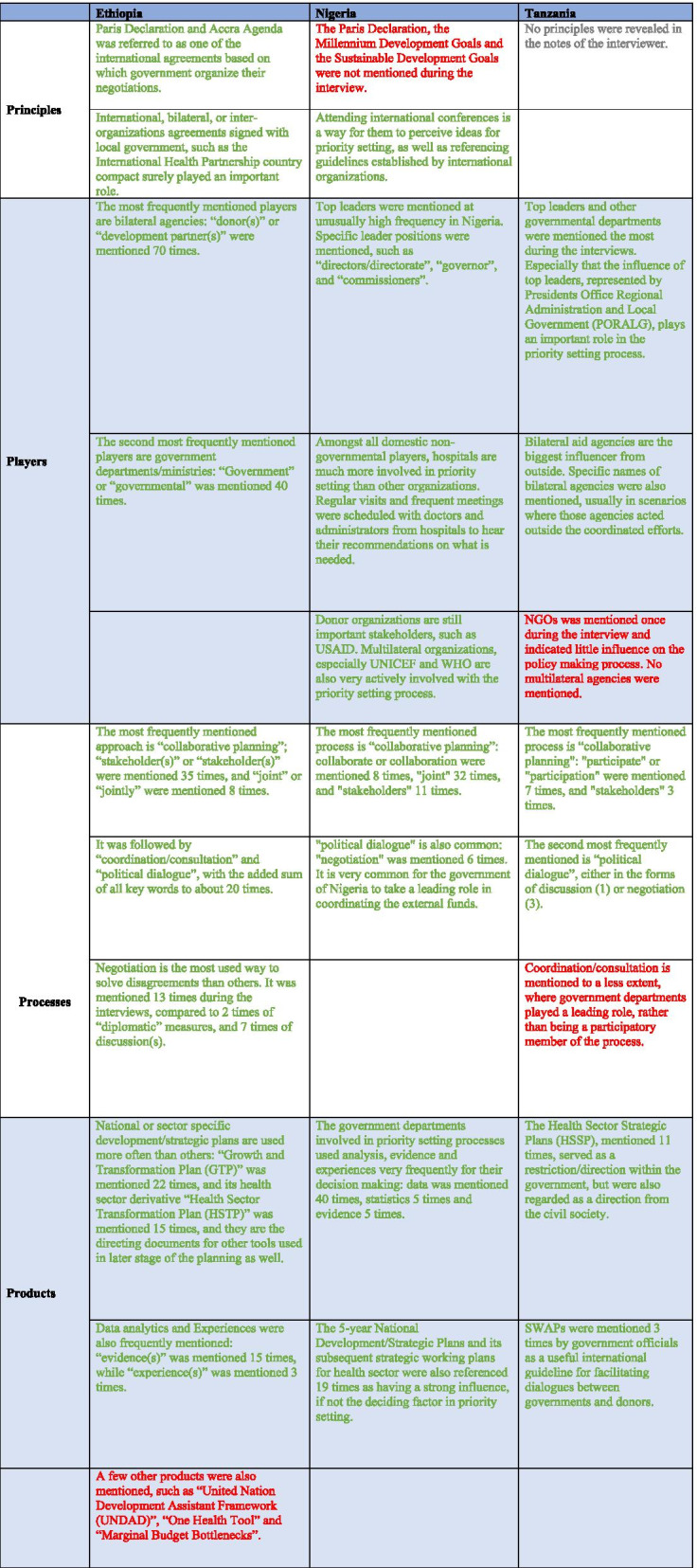
Themes mentioned by government officials in Ethiopia, Nigeria, and Tanzania, for the priority setting process

Factors with major influence were marked in green, while factors with minor or no influence were marked in red

### Principles of guidance

Limited data emerged from the interviews conducted in Tanzania relating to the analysis of principles. Richer data enabled a comparison of Ethiopia and Nigeria on the principles that were used in the priority setting process for ODA. Analysis revealed that International agreements, including Paris Declaration and Accra Agenda, as well as International Health Partnership country compact played important roles in the priority setting process in Ethiopia:

*“…Based on different international declaration such as the Paris and Accra declarations the government negotiates by prioritizing the national interest.” (one government official from Ministry of Finance and Economic Development (MoFED), Ethiopia)*

*“Since different agreements are signed between the funder and the government, disagreements are less to happen.” (one government official from MoFED, Ethiopia)*

By contrast, none of those principles were mentioned by interviewees in Nigeria. Instead, different officials agreed that attending international conferences was a way for them to perceive ideas for priority setting, as well as reading guidelines established by international organizations:

*“The federal ministry of women affairs normally organizes council meetings, in that place we prepare a lot of proposals and prepare for what we'll do the coming year. Aside from that, every March, they normally attend the U.N status of women in New York. So, they come with new things by that time based on global focus.” (one government official from* the *Ministry of Women Affairs and Social Development, Nigeria)*

*“Another thing is we can look at the WHO guideline and seek for approval along with this guideline that is if the policy is domesticated and accepted in Nigeria” (one government official in charge of Communicable Diseases, HIV/AIDS, and TB [tuberculosis], Nigeria)*

### Players involved

The most frequently mentioned players in Ethiopia and Tanzania were bilateral agencies: “donor(s)” or “development partner(s)”, followed by government departments/ministries. The context in which bilateral agencies were mentioned depicts a spectrum of roles these agencies were playing in priority setting. In most cases, they were mentioned together with other stakeholders as a member of the collaborative planning process:

*“…Based on the revised policy the government sets sector-based plans and in consultation with different stakeholders including donors.” (one government official from MoFED, Ethiopia)*

*“HSHSP [Health Sector HIV and AIDS Strategic Plan] IV includes both GOT [Government of Tanzania] and donor priorities as it is developed in a participatory manner.” (one government official from Ministry of Health, Community Development, Gender, Elderly and Children (MOHCDGEC), Tanzania)*

In other cases, bilateral aid agencies acted alone, but were also following the national plan of local governments:

*“So based on our Growth and Transformation Plan, donors will provide support by aligning with the nationally set priorities and local agendas.” (one government official from MoFED, Ethiopia)*

A few scenarios were mentioned when conflicts arose between local governments and donor organizations:

*“the donor may request to use its own consultant to work different activities while the Ministry can do it with the available staff, this causes a conflict of interest between the two.” (one government official from MoFED, Ethiopia)*

In Nigeria, however, top leaders were mentioned with unusually high frequency. Specific leader positions were mentioned, such as “directors/directorate”, “governor”, and “commissioners”. In Tanzania, top leaders, represented by Presidents Office Regional Administration and Local Government (PORALG), also played an important role in the priority setting process:

*“But, in the Ministry of Health which happens to be the mother of all related agencies, so, it is the Director of Administration and Supplies, they are saddled with that exercise. He is the one that will write a memo regarding that and make a request.” (one government official from Oyo State Ministry of Health, Nigeria)*

*“For instance, the Ministry of Health has been able to convince the permanent secretary and the commissioner for health that we need this much resources to do something.” (one government official from the Malaria Program, Nigeria)*

Hospitals in Nigeria were much more involved in priority setting than other organizations. Regular visits and frequent meetings were scheduled with doctors and administrators from hospitals to hear their recommendations on what is needed:

*“Not only that, but we also meet with our doctors in our various hospitals once every month, first Friday to be precise. It is a technical meeting where they give us a report of what has happened in the various hospitals in the last month. They help us know the disease pattern, know the area we need to focus and give us the ability to compare the hospitals or facilities where there’s health insurance with facilities or hospitals where there is no health insurance.” (one staff from Oyo State Health Insurance Agency)*

Donor organizations were identified as important stakeholders in Nigeria, such as the United States Agency for International Development (USAID), in the priority setting process but to a much lesser extent compared to Ethiopia and Tanzania.

Interview findings from all three country cases indicated that non-governmental organizations (NGOs) and multilateral agencies had little direct influence over the priority setting process. However, they could influence other elements of the framework, for example the Processes utilized and the Products used, as described below.

### Processes utilized

In Ethiopia, Nigeria, and Tanzania “collaborative planning” was the most frequently mentioned process in priority-setting, and involved multiple stakeholders, each of which played a relatively equal role in decision making:

*“All of the mentioned stakeholders will also participate in decision making and agenda prioritization but the main role in agenda-setting is led by FMOH.” (one government official from Federal Ministry of Health (FMoH), Policy and Planning, Ethiopia)*

*“They always bring their work plan which at times may not suit our challenges. But this time around, both of us will sit down and look at their work plan and look at our work plan and we merged them.” (one government official from Oyo State Ministry of Health, Nigeria)*

*“Annual stakeholders meeting is a forum for all partners, implementers and beneficiaries to discuss issues related to TB and Leprosy including the sharing of annual operational plans.” (one government official from National TB and Leprosy Programme (NTLP)-MOHCDGEC, Tanzania)*

“Political dialogue and “coordination/consultation” initiated by local governments were also quite commonly used processes in all the three countries studied. In Ethiopia, political dialogue usually took the form of negotiation, while in Nigeria, it was mentioned as meetings and discussions, and in Tanzania, as discussions and negotiations:

*“During setting priorities, the government takes the lead however consultations are commonly requested from different stakeholders to finalize and approve priorities.” (one government official from MoFED, Ethiopia)*

*“…the major problem is the vacuum called ‘meeting, discussion’ before the final approval of the project.” (one government official from the Ministry of Women Affairs and Social Development, Nigeria)*

*“MOHCDGEC and PORALG had different views on this issue and through dialogue, a consensus was reached.” (one government official from Directorate of Policy and Planning (DPP)-MOHCDGEC, Tanzania)*

*“Dialogue is the approach used to address any disagreements. A good example is when the PORALG wanted to use the balance from unused HBF [Health Basket Funds] funds for upgrading or putting up a new infrastructure for health facilities. The Sector ministry and Donors had a different opinion, but after repeated dialogue, all parties agreed to use the funds for an infrastructure upgrade. (one government official from PORALG, Tanzania)*

Political power was also used in the priority setting process. It included diplomatic power, as well as power to influence higher levels of government hierarchy and judiciary power that imposed legal constraints on certain behaviors:

*Conflicts that arise mostly due to implementation (if the project implementation deviates from the directive and proclamation), are primarily resolved by negotiation. If the dispute is not resolved by negotiation, then it will be referred to be handled by the federal court. (one official from the Social Charity organizations, Ethiopia)*

“Conditioned financial support” was mentioned in all three cases, suggesting that donor influence through funding was still very prevalent:

*“The first word I will say that he who has the piper dictates the tune. No matter what your priorities are, by the time someone is bringing money to help you, but then these are the ways at which you can go, the best you can do is to see how well their most mode of operation will end up suiting your own expectation.” (one government official from Oyo State Ministry of Health, Nigeria)*

*“Partners would in many cases come up with innovations that are not included in HSHSP IV and would like to fund pilots.” (one government official from National AIDS Control Program-MOHCDGEC, Tanzania)*

### Products used

In all three cases, national or sector-specific development/strategic plans were the most frequently used product in guiding the priority setting process. These products were variously called “Growth and Transformation Plan (GTP)” and “(5- year) Health Sector Transformation Plan (HSTP)” in Ethiopia, “5-year National Development/Strategic Plans” in Nigeria, and “Health Sector Strategic Plan (HSSP)” in Tanzania:

*“Currently there are around 28 United Nations (UN) agencies. These agencies have a framework for budgeting and implementing planned activities. United Nations Development Assistant Framework (UNDAF) is a framework which will be signed by all UN agencies and all are under this document. Using this framework country-specific projects will be designed for five years based on the sector plan or GTP, for UNICEF the plan will be following the HSTP.” (one government official from Multilateral collaboration, MoFED, Ethiopia)*

*“there must be a memorandum of understanding, this MOU is review by the desk officer to see if it goes with what is (in) the strategic plan.” (one government official in charge of Communicable Diseases, including HIV/AIDS, TB, Nigeria)*

*“The HSSP guide the sector and all stakeholders. It is a promise to the public by the Government.” (Notes from a policy consultant with the Government of Tanzania for the interview with a government official from DPP-MOHCDGEC, Tanzania)*

In Ethiopia, priority setting took a “cascade”-like hierarchical process, where each level of priority needed to be set based on or following the agreement or principles finalized by upper-level government bodies:

*“Five-year strategic plan is set and prioritized issues including resource gaps are addressed in the strategic plan. The mother document is the GTP and every other thing evolved from this document.” (one official from FMoH, Ethiopia)*

Data analytics and Experiences were also frequently mentioned in both Ethiopia and Nigeria, which included epidemiological data, demographic data, and among others, economic analysis:

*“Disease burden is reviewed during planning, including mortality, morbidity, impact on the economy and also human right issues, then alternative interventions will be proposed and finally will be prioritized.” (One government official from FMoH, Resource mobilization, Ethiopia)*

*“Zamfara may tell you they have understood there are seasonal variations… I cannot attempt that in Oyo State. Because I know malaria in my state is year-round.” (one government official from the Malaria Program, Nigeria)*

In Tanzania, SWAPs were mentioned multiple times by government officials as a useful international instrument for facilitating dialogues between governments and donors:

*“Tanzania has a functioning SWAP dialogue structure that puts in use the above.” (one government official from DPP-MOHCDGEC)*

### Main findings from the cross-country comparative analysis

Cross country analysis revealed similarities as well as differences relating to Principles guiding priority setting decisions, the Players involved, the Processes utilized, and the Products used.

First, there was noticeable variation in the Principles used and Players involved in the priority-setting process in Nigeria, a lower-middle-income country where external funding accounted for less than 10% of the current total health expenditure, as compared with Ethiopia and Tanzania, both low-income countries, where external funding accounted for 22 and 32% of the total health expenditures respectively.

In Nigeria, Principles appeared in more informal ways, for example the principles garnered from attending international conferences. While in Ethiopia, formal principles, such as the Paris Declaration, the Millennium Development Goals, or the Sustainable Development Goals, as well as signed agreements under the International Health Partnership were mentioned as their guiding principles. In Nigeria, local government, especially the top leaders took a more direct role in setting priorities in the health sector. However, in both Tanzania and Ethiopia, bilateral aid agencies were mentioned most frequently when asked to describe the priority setting process. Non-governmental organizations remained as very marginalized players in decision-making related to resource allocation in health.

In all the three countries there was more convergence with Processes and Products. Across the three study countries: 1) collaborative planning was the most commonly used process, and 2) the Health Sector or National Strategic Plan were the most commonly utilized products in the priority-setting process.

The findings suggest that multiple stakeholders working around one national plan was how priorities were established in these countries. Political dialogue was also frequently used by all three countries as a process to reach an agreement with other stakeholders and/or to resolve an existing conflict of ideas. This took the form of discussions, meetings, and negotiations.

In addition to Health Sector Plans of Strategies, evidence and experiences were two other products used in setting health priorities, more prominently in Ethiopia and Nigeria than that in Tanzania.

There were minor differences in the processes and products used across the three case countries: consultation and coordination were mentioned more often in Ethiopia, where the government assumed a more leading role were mentioned, than in Nigeria and Tanzania. In Ethiopia, there were also other products mentioned as useful tools: “United Nations Development Assistant Framework”, “One Health Tool” and “Marginal Budget Bottlenecks”. In Tanzania, Sector Wide Approaches were mentioned multiple times.

## Discussion

We developed a new analytical framework to explore priority setting processes in health and tested it with primary qualitative data from key informant interviews undertaken in Ethiopia, Nigeria, and Tanzania. We conducted a comparative analysis to understand which principles were used to guide the priority setting process for ODA, the extent of engagement by different players, and the application of different processes, and products.

The analytic framework was developed to examine priority setting as a dynamic process where each component of the framework interacts with each other to shape decisions. Many priority setting tools and approaches have been developed over the past decades, including cost-effectiveness analysis and the extended cost-effectiveness analysis [41], burden of diseases analysis and etc. Others have argued for a multi-criteria decision analysis [42]. For example, Shiffman and Smith have proposed a similar framework for priority setting, where they listed four categories as actor power, ideas, political contexts, and issue characteristics [43]. This paper contributes to the latter line of thought, with an emphasis that we should not view the process as selecting from a “laundry list” of interventions for the health sector [44] but should regard it as a process in a complex dynamic system. Our paper contributes to the scholarly efforts in applying systems thinking in health systems and the framework developed and tested in this article can be used to pave the way forward to make systems thinking a more practical tool in health system analysis. In addition, the Shiffman and Smith framework takes the donor perspective, while our framework was both built and tested using data from the perspective of local governments on the receiving end of the ODA.

Our data also showed a few specific lessons to be learnt for priority setting for ODA in LMICs. Findings from the three case studies revealed that donors and top government leaders still remained the most influential players in priority setting for ODA and in the health sector in general, and involvement of civil society organizations was very low. Our findings corroborated those from earlier studies [45] which demonstrated that public engagement remained elusive despite active attempts from donors and recipient governments to include civil society in decision-making processes.

Comparative analysis across the three country cases seemed to suggest that in countries where a higher proportion of health expenditure is from external sources, donor agencies tended to have stronger influence in the priority setting process. Recent case studies from research undertaken in Cambodia and Pakistan [46] explained how donors influence the agenda setting of recipient countries, mostly through their control over direct and indirect financial and political influences – a finding also revealed by our study.

Collaborative planning and political dialogue were the most commonly used process by local governments, with consultation used to a lesser extent. This finding is in line with various models proposed for the priority-setting process [47–49] which emphasizes inclusiveness, transparency and broader stakeholder engagement. Two other processes could be used by recipient governments to enhance the priority setting process and ensure a more balanced approach. First, actively identify international recommendations that support their arguments on why certain areas should be included as priorities. Second, establish strong local coalitions, engage multiple sources of political power including higher-level government leaders, draw on the legal and regulatory limits set by other government departments, and make use of public opinion.

National strategic plans for health have been recommended by the World Health Organization [50] as a useful tool in establishing health priorities, while country level implementation is not always satisfactory [51]. Our case studies revealed that all three countries used a national strategic plan as the guiding product for all levels of decision making. The national strategic plan was the most frequently mentioned product among all others used in our case countries, and it took a collaborative process that included multiple stakeholders where the local government seemed to take a more leading role: “*It is a promise to the public by the Government*”, as one Tanzanian government official put it. However, we are also aware of, and our data shown that aid agencies have multiple channels of influence to alter the content of strategic plans if needed, such as through the use of conditioned financial support. The analysis of primary data revealed how the four elements of the framework, Principles, Players, Processes, and Products, interacted with each other, to reinforce and sometimes to counter effect to influence and shape the decision making for priority setting for ODA.

Use of data can be a highly effective tool in persuading other stakeholders to agree with the national plan: local governments can invest in data-generating skills within the organization or with partners. Past experiences are also powerful in influencing the design and implementation of health actions, especially where data are either scarce or too simplistic for real-world applications. Local government could make better use of past experiences by establishing a good recording system of what happened, whether an intervention or policy worked or not, and why.

In summary, data from the case studies support the existence of all four layers in the a priori framework and their interaction. However, not all the three countries were actively using all the elements within each layer in their priority setting process for ODA, which suggests a large unfulfilled potential to build and expand country-level alliances. In particular, in relation to the Players, many country-level institutions were currently underutilized, including legal and regulatory departments within the government, other domestic players and multilateral agencies, and international entities.

Upon reviewing and reflecting on the primary data emerging from the key informant interviews and the context of the extracted themes, the authors also identified a few places where the framework could be further strengthened and improved. First, processes and products have overlapping functions, for example, the Swaps was referred to as a “process” or an “approach” in the transcripts. Second, there is association of certain themes across the four layers, for example, the United Nations agencies, one of the players frequently involved in decisions pertaining to priority setting for ODA, usually have specific processes to follow in their interactions with recipient countries that lead to the development of products. Another example of such an association is that the development of health sector strategic plan, a frequently used product, often involved the process of collaborative planning to build. Third, although the framework depicts the major components of a dynamic and interconnected system related to decision making for priority setting, it does not reveal how the system works, for example, through different feedback loops and generating non-linear effects. More research is required, rooted with the systems theory, to further explore the validity and generalizability of this framework in relation to the interactions among the components of the framework.

The COVID-19 pandemic has presented a series of major challenges to the health systems in LMICs [52]. The response to COVID-19 is consuming large amounts of public health resources with substantial negative impact on existing health delivery programs, especially for vulnerable populations including women and children [53, 54]. In the foreseeable near future, priority setting process for health in LMICs will continue to be swayed towards funding actions and interventions to mitigate and combat health threats and emergencies, rather than efforts in maintaining or expanding routine health programs aimed at addressing other health needs. Decision makers are in more need of better tools in making sure resources are allocated in just, efficient and sustainable manner to locally relevant needs.

Our study has limitations. The small sample size of the key informants interviewed in each country might render the data incomplete and thus may miss other key features that might be revealed by more interviews. In addition, interviews were conducted in Amharic in Ethiopia and Swahili in Tanzania, but only the English translation of those transcripts/notes were analyzed. This means that some intricate meanings in between the lines might be lost during the translation. Further, the study did not explore in depth the interactive nature of each component of the framework, for example the correlation of use of certain processes with higher power presented by local governments or bilateral agencies, or the association of active engagement of certain players with more power to the local government in collaborative planning. However, notwithstanding the limitations, the study reveals novel findings and identifies new questions for future research that could help answer them, and provides a new framework for analysis.

## Conclusions

Our analysis reveals that in the countries analyzed, health strategies and priorities are not made by selecting from a list of options available to decision-makers, but it is a dynamic process in a complex system that involves multiple pathways that interact with each other and each pathway and their interaction contribute to the outcome in its own way.

This article presented how a novel framework can be used in analyzing the priority setting process in countries that receive ODA and in comparative analysis. The analytical framework enabled the identification of shared features, as well as variances in priority setting process across countries.

The comparative analysis has provided insights into what resources and strategies local governments could utilize to ensure greater engagement and more prominent role in priority setting to balance donor power and improve the effectiveness of the official development assistance for health.

## Data Availability

The datasets used and/or analysed during the current study are available from the corresponding author on reasonable request.

## Abbreviations

AIDs: Acquired Immunodeficiency Syndrome
DPP: Directorate of Policy and Planning
FMoH: Federal Ministry of Health
GOT: Government of Tanzania
GTP: Growth and Transformation Plan
HBF: Health Basket Funds
HIV: Human Immunodeficiency Virus
HSHSP: Health Sector HIV and AIDS Strategic Plan
HSSP: Health Sector Strategic Plan
HSTP: Health Sector Transformation Plan
IHP+: International Health Partnership Plus
LMICs: Low- and Middle-Income Countries
MDGs: Millennium Development Goals
MoFED: Ministry of Finance and Economic Development
MOHCDGEC: Ministry of Health, Community Development, Gender, Elderly and Children
MOU: Memorandum of understanding
NGOs: Non-governmental organizations
NTLP: National TB and Leprosy Programme
ODA: Official Development Assistance
PORALG: President’s Office Regional Administration and Local Government
PORALG: President’s Office Regional and Local Government
SIPs: Sector Investment Plans
SWAPs: Sector Wide Approaches
TB: Tuberculosis
UN: United Nations
UNDAF: United Nations Development Assistant Framework
USAID: United States Agency for International Development
WHO: World Health Organization
